# Statistics of Consumption

**Published:** 1846-11

**Authors:** 


*Statistics of Consumption.*—Dr. Theophilus Thompson gave a short report to the Medical Society of London, of some particulars which he had observed, during the last twelve months, as visiting physician to the Hospital for Consumption and Diseases of the Chest. The number of patients treated by him during the year was 760, of which 286 were phthisis in various degrees of advancement. Amongst 77 cases of advanced phthisis, 56 were men, only 22 women; but of the cases of incipient phthisis, the number of males and females was nearly equal—a fact leading to the conclusion that the apparent preponderance of the former was attributable to the unwillingness or inability of women to leave their homes under circumstances of advanced disease. He remarked on the importance of prolonged expiratory murmur, when unconnected with bronchitis or emphysema, as an early indication of phithisis, and a sign which, when once established, rarely disappears. He also particularly noticed, as a phenomenon of great interest and practical importance, the "inspiration saccadée" of some French authors—not the jerking respiration of spasmodic asthma, nor the interrupted inspiration of diffused pleurisy, but the division of the inspiratory murmur, as though the entrance of the air into the cells required several successive efforts. He had occasionally observed this sign at the back as well as the front part of the chest. It sometimes disappeared under treatment; but there was reason to think it characteristic of a condition of the lungs which frequently immediately preceded, or accompanied, tubercular infiltration. It was remarkable, that of ten cases recorded during the year, the phenomenon had been in nine cases confined to the left side. He had, during the last twelve months, taken notes of eight cases in which a murmur was heard in the second intercostal space, on the left side only, and was probably referable to the pulmonary artery. In two of these patients, the murmur disappered under the use of iron; but in most it was succeeded by more or less distinct manifestations of tubercular disease. He deferred any comments on cases of heart-disease, bronchitis, and other pectoral affections, and concluded by mentioning the results of his observations regarding cod-liver oil, which he had administered in 37 of the recorded cases. In 3, the medicine was discontinued in consequence of the distressing nausea which it occasioned; in 12, the reduction of strength appeared to be slightly retarded; in 12, there was no perceptible effect; in 10, the increase of strength, plumpness, and energy was remarkable. When the fattening process was established it generally became obvious within a fortnight. The author did not attribute to the oil any specific influence on the local disease; but believed it to be singularly efficacious in promoting nutrition. He had found it most useful to the pallid and phlegmatic, and, in private as well as public practice, had observed more decided amelioration under its employment than could be referred to any other remedial means with which he was conversant.—*London Lancet.—Boston Med. and Surg. Jour.*

